# Preparation of size-tunable sub-200 nm PLGA-based nanoparticles with a wide size range using a microfluidic platform

**DOI:** 10.1371/journal.pone.0271050

**Published:** 2022-08-04

**Authors:** Yi Bao, Masatoshi Maeki, Akihiko Ishida, Hirofumi Tani, Manabu Tokeshi

**Affiliations:** 1 Graduate School of Chemical Sciences and Engineering, Hokkaido University, Hokkaido, Japan; 2 Division of Applied Chemistry, Faculty of Engineering, Hokkaido University, Hokkaido, Japan; 3 JST PRESTO, Kawaguchi, Japan; Osaka Shiritsu Daigaku, JAPAN

## Abstract

The realization of poly (lactic-co-glycolic acid) nanoparticles (PLGA NPs) from laboratory to clinical applications remains slow, partly because of the lack of precise control of each condition in the preparation process and the rich selectivity of nanoparticles with diverse characteristics. Employing PLGA NPs to establish a large range of size-controlled drug delivery systems and achieve size-selective drug delivery targeting remains a challenge for therapeutic development for different diseases. In this study, we employed a microfluidic device to control the size of PLGA NPs. PLGA, poly (ethylene glycol)-methyl ether block poly (lactic-co-glycolide) (PEG-PLGA), and blend (PLGA + PEG-PLGA) NPs were engineered with defined sizes. Blend NPs exhibit the widest size range (40–114 nm) by simply changing the flow rate conditions without changing the precursor (polymer molecular weight, concentration, and chain segment composition). A model hydrophobic drug, paclitaxel (PTX), was encapsulated in the NPs, and the PTX-loaded NPs maintained a large range of controllable NP sizes. Furthermore, size-controlled NPs were used to investigate the effect of particle size of sub-200 nm NPs on tumor cell growth. The 52 nm NPs showed higher cell growth inhibition than 109 nm NPs. Our method allows the preparation of biodegradable NPs with a large size range without changing polymer precursors as well as the nondemanding fluid conditions. In addition, our model can be applied to elucidate the role of particle sizes of sub-200 nm particles in various biomedical applications, which may help develop suitable drugs for different diseases.

## Introduction

Over the past decades, polymeric nanoparticles (NPs), especially biodegradable polymers, have emerged for building a drug delivery system (DDS) [1–3]. NPs comprising poly(lactic-co-glycolic-acid) (PLGA) have been widely used as carriers for hydrophilic and hydrophobic drugs, as well as proteins, vaccines, and siRNA [4–7], owing to their excellent biodegradability and biocompatibility [8]. By employing PLGA, countless laboratory synthesis methods present novel PLGA-based NPs targeting and inhibiting cancer cells; nevertheless, only a minority of formulations have achieved clinical translation and effects on humans [9–12]. To some extent, the challenge lies with the complexity of NP optimization. For every disease type, it is essential to find the optimal physicochemical parameters (such as particle size, surface charge, morphology, and rigidity) and assess tissue targeting, designed drug release, and immune evasion.

In KB carcinoma cell lines, 70 nm-sized PLGA NPs took up more than 200 nm-sized PLGA NPs, whereas, in RAW264.7 macrophages, 70 nm-sized NP engulfment was less than 200 nm-sized NPs [13]. In two human colon cancer cell lines (Caco-2 and HT-29 cells), the cellular uptake rate of 100 nm-sized didodecyldimethylammonium bromide (DMAB)-modified PLGA NPs was higher than that of 50 nm-sized NPs [14]. The uptake of 100 nm particles in Caco-2 cell lines was 2.3 times greater than that of 50 nm-sized NPs [15]. These results indicate that NPs sized approximately 100 nm have a profound size effect (from 50 nm to 200 nm). Precise preparation of particle-size-controlled NPs is of great significance for establishing different targeted drug delivery systems. Particle size is a crucial feature in the DDS design, which determines the in *vivo* distribution, cytotoxicity, and stability of NPs and influences drug loading and release [16]. The particle size can control the particle distribution in the body to some extent [4, 17, 18]. Therefore, preparing polymeric NPs with a controllable size is critical to precisely control drug loading and release and, more importantly, realizing building size-targeted DDS. To extensively investigate various disease types, a wide range of size-controlled PLGA-based NPs at the nanoscale level is imperative. Generally, to approach size-controlled PLGA NPs with a broad size range, researchers tend to change the polymer precursors (polymer composition, concentration, and molecular weight (Mw)). However, changes in precursor condition would interfere with NP characteristics, such as drug loading ability, cytotoxicity, and degradability. Thus, it is of great significance to prepare size-customized PLGA-based NPs with a broad size range without varying the precursors, which could meet the different demands of NPs.

In contrast to conventional PLGA NPs preparation methods, such as emulsification–solvent evaporation, spray-drying, and phase separation, a microfluidic method produces size-controlled NPs with a narrow size distribution and good size batch-to-batch reproducibility [19–21]. Furthermore, using a solvent that is rapidly diluted with an aqueous solution in a microfluidic device, through varying flow conditions in microscale channels, enables the manipulation of nanoliter volumes, and the physicochemical features of the NPs can be controlled. In summary, the microfluidic method has enormous potential for substantiating size-controlled PLGA-based NPs.

At present, PLGA NPs with size control have been prepared using microfluidic methods [20–23]. However, size control over a wide range is achieved by changing the type of precursor. Unlike the nanoprecipitation method, Karnik *et al*. used a flow-focusing microfluidic device to mix miscible polymer solutions rapidly with water, and copolymers could self-assemble into formate NPs. By adding PLGA to poly (ethylene glycol) methyl ether block poly (lactic-co-glycolide) (PEG-PLGA) in different amounts, NPs were prepared in the range of 30–105 nm [23]. Rhee *et al*. produced NPs with the average particle size of 30–230 nm mainly by modulating the molecular weight (27, 45, and 95 kDa) and varying concentration of PLGA from 10 to 50 mg/mL [20]; Valencia *et al*. designed devices with Tesla structures in microchannels to prepare NPs with the average particle size of 35–180 nm by changing the PLGA and DSPE-PEG composition and concentration of precursors [21]. If preparing a large range of size-controllable NPs without changing the polymer precursors is not achievable, finer customization, including the specified PLGA compound composition and controlled size of PLGA NPs, could not be allowed. To the best of our knowledge, no studies have been reported on manipulating the size-customized sub-200 nm PLGA-based NPs over a broad size range and with a good batch-to-batch repeatability without varying precursors.

**Fig 1:**
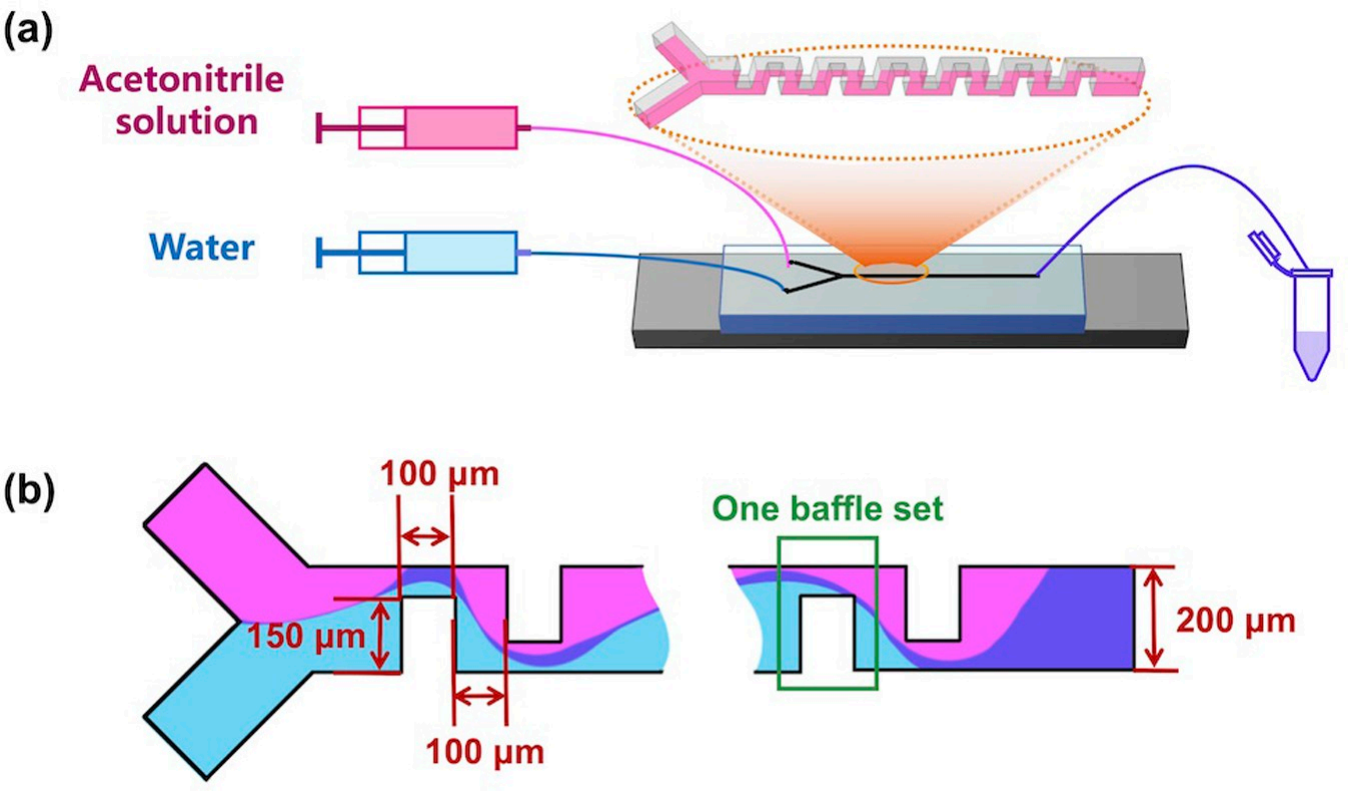
(a) Schematics of the iLiNP device to prepare blank or PTX-loaded NPs; (b) Top view of the iLiNP device. The width and depth of the channel were 200 and 100 μm, respectively. The device was equipped with 20 baffle sets.

In this study, we employed iLiNP^®^, designed by our group [24], to prepare a considerably wide variable nanoscale size range of PLGA-based NPs, without changing the polymer composition. Fig 1 shows a schematic of the iLiNP device. The prepared PLGA NPs exhibited excellent reproducibility and narrow size distribution. Owing to its latent capacity for medical application, paclitaxel (PTX) was employed as a model cancer therapy drug to be delivered with a high encapsulation efficiency (EE). Alternately, the cytotoxicity of different-sized PTX-loaded NPs *in vitro* was also investigated, which proved the potential capacity of the same composition PLGA size-controlled NPs within a wide size range in future DDS medical research.

## Materials and methods

### Materials

PLGA (Mw = 24,000–38,000) and PEG-PLGA (average *Mn*_PEG_ = 2,000, average *Mn*_PLGA_ = 11,500) were purchased from Sigma–Aldrich (St. Louis, MO, USA). PTX was purchased from Tokyo Chemical Industry, Ltd. (Tokyo, Japan). Acetonitrile for the dissolving solvent and high-performance liquid chromatography (HPLC) was supplied by FUJIFILM Wako Pure Chemical Corporation (Osaka, Japan).

### Fabrication of microfluidic devices

The design of the iLiNP device is illustrated in Fig 1. The width and depth of the channel were 200 μm and 100 μm, respectively. The polydimethylsiloxane (PDMS)-based iLiNP device was fabricated, followed by standard soft lithography [22], and bonded with a glass substrate to compose the iLiNP device. The detailed fabrication procedure of the iLiNP device has been reported previously [24].

### Preparation of PLGA-based nanoparticles

The polymer (PLGA, PEG-PLGA, or blend [PLGA mixed with PEG-PLGA] with a mass ratio of 1:1) was dissolved in acetonitrile at different concentrations of 5 mg/mL. Then, PLGA/acetonitrile solution and ultrapure water (Milli Q; Direct-Q UV system, EMD Millipore Corporation, Billerica, MA, USA) were introduced into the iLiNP device from the two inlets. Two glass syringes (GASTIGHT 1002; Hamilton Inc., Reno, NV, USA) were filled with a polymer solution and ultrapure water, respectively. The syringes were connected to a microfluidic device, and syringe pumps (LEGATO 210; KD Scientific Inc., Holliston, MA, USA) were used to feed the solutions into the microfluidic device. After collecting the NPs in a microtube from the outlet, the solution was dialyzed in ultrapure water using a membrane tube (MWCO: 12–14 kD; Spectrum Laboratories, Inc., Canada) overnight at 4°C. Finally, the size and stability of the NPs were evaluated using dynamic light scattering (DLS, Zetasizer Nano ZS ZEN3600; Malvern Instruments, UK).

For the conventional method, polymer (PLGA, PEG-PLGA, or blend [PLGA mixed with PEG-PLGA] with a mass ratio of 1:1) was dissolved in acetonitrile at 5 mg/mL concentration, and the polymer solution was added to ultrapure water using a micropipette with stirring. The NP suspension was dialyzed overnight against ultrapure water.

When prepared using the chaotic mixer device, the method is similar to that of iLiNP. Briefly, the polymer (PLGA, PEG-PLGA, or blend [PLGA mixed with PEG-PLGA] with a mass ratio of 1:1) was dissolved in acetonitrile at a concentration of 5 mg/mL. The acetonitrile solution and ultrapure water in two syringes were then injected into the chaotic mixer device through two inlets using a syringe pump. The solution collected from the outlet would be dialyzed overnight in ultrapure water by the membrane tube.

### Preparation of PTX-loaded nanoparticles and determination of drug content

Similar to the empty PLGA NPs, 5 mg/mL polymer and 0.5 mg/mL PTX (10% of polymer) were dissolved in acetonitrile, followed by introduction into the iLiNP device with ultrapure water from two different inlets. To remove the organic solvent and non-encapsulated drug, the collected solution was dialyzed overnight through the membrane tube in the ultrapure water. The PTX EE was determined using HPLC (L-2000 Elite LaChrom HPLC system; HITACHI, Japan). The NP solution was freeze-dried using freeze-drying equipment (Tokyo Rikakikai Co., Ltd., Japan), and the powder was dissolved in acetonitrile to dissolve the polymer and loaded PTX. The solution was filtered for HPLC analysis. A reverse-phase column (Shodex C18M 4D [inside diameter 150 × 4.6 mm, pore size 5 μm]; Shodex, Japan) was used to separate polymers and PTX, and the column temperature was maintained at 30°C. The mobile phase consisted of acetonitrile/water (50:50 v/v) at a flow rate of 1.2 mL/min. PTX concentration was measured at a wavelength of 227 nm, and 20 μL of the sample was injected using an autosampler. PTX solutions ranging from 5 to 100 μg/mL were prepared to construct a calibration curve. PTX EE was defined as the ratio of the amount of drug in the NPs to the initial amount of drug used for the preparation (Eq (1)).


$$\text{Encapsulation}\;\text{Efficiency}(\text{EE}\%)=\frac{\text{Amount}\;\text{of}\;\text{PTX}\;\text{in}\;\text{nanoparticles}}{\text{The total}\;\text{amount}\;\text{of}\;\text{PTX}}\times 100\%$$ (1)

### Cytotoxicity studies

HeLa cells (kindly gifted by Dr. Yusuke Sato at Hokkaido University) were cultured in Dulbecco’s modified Eagle’s medium (DMEM, Sigma–Aldrich) supplemented with 10% fetal bovine serum (FBS) and 1% penicillin–streptomycin in an incubator with an atmosphere of 5% CO_2_ and 37°C. Blend-PTX NPs were prepared under concentration of 5 mg/mL blend (PLGA mixed with PEG-PLGA) with a mass ratio of 1:1 and 0.5 mg/mL PTX (10% of polymer). Two different sized blend-PTX were prepared with the flow condition as TFR is 50 μL/min or 500 μL/min, while the FRR is 3. After obtainting the EE of the NPs, blend-PTX NPs were diluted to solutions of five different PTX concentrations by medium for cell uptake studies. Cytotoxic activity was evaluated using CellTiter-Blue (Promega, US) cell viability assay, and the steps are shown below.

Cells were seeded at a density of 5000 viable cells/well in 100 μL of the medium in a black 96-well microplate (Nunc, Denmark). The cells were then incubated with different concentrations of PTX in the medium solution or with different concentrations of PTX-loaded NPs. The microplates were incubated for 1, 2, or 3 d. After incubation, 20 μL CellTiter-Blue were added to each well, and the microplates were further incubated for 2 h. The fluorescence signal was measured using a fluorescence microplate reader at 560_ex_/590_em_ nm. Cell viability was determined using Eq (2)

$$\text{Cell}\;\text{viability}(\%)=\frac{{\text{A}}_{\text{sample}}-{\text{A}}_{\text{negative}}}{{\text{A}}_{\text{positive}}-{\text{A}}_{\text{negative}}}\times 100\%$$ (2)

where A_sample_, A_negative_, A_positive_ are the fluorescence intensities of the sample, the negative control, and the positive control, respectively.

### Statistical analysis

The result are expressed as mean ± standard deviation and analyzed using ANOVA to demonstrate statistical differences. The predictive value (P) ≤ 0.05 was considered statistically significant.

## Results and discussion

### Effect of the flow conditions on NP size

**Fig 2:**
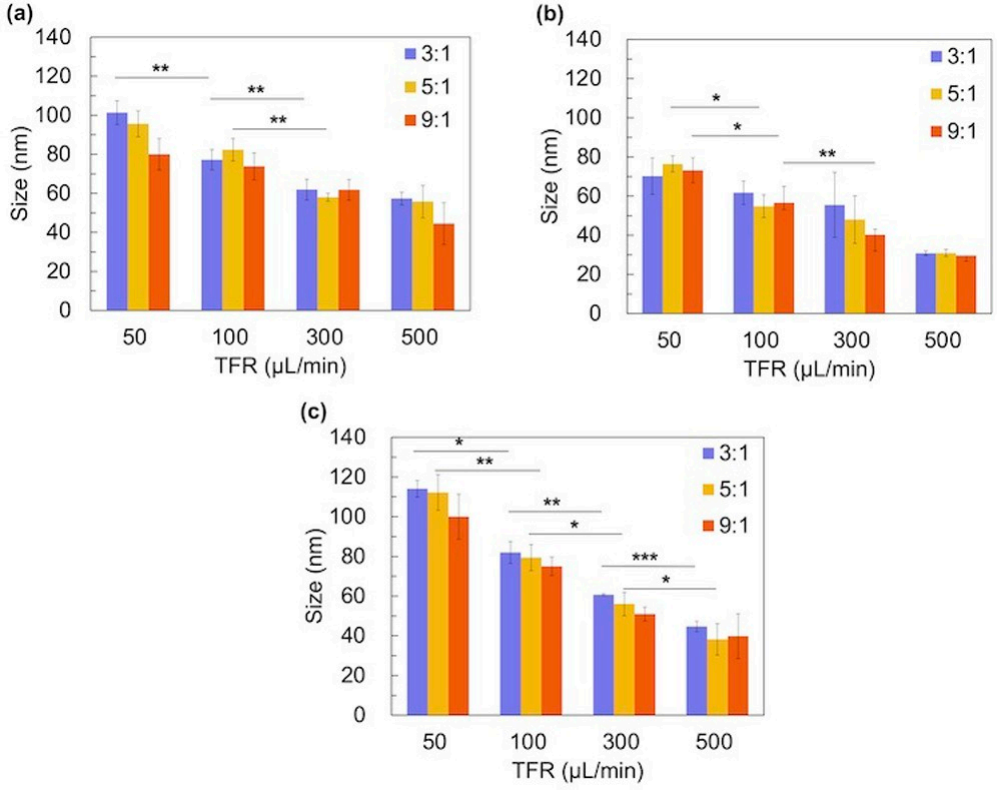
Effect of the flow condition on the NP size. (a) PLGA, (b) PEG-PLGA, and (c) blend. The FRR ranges from 3:1 to 9:1, whereas TFR ranges from 50 to 500 μL/min. The error bars represent the standard deviations calculated from the repeated NP preparation experiments at least three times. P-values: ***≤0.0001; **≤0.01, *<0.05.

To investigate the effect of flow conditions on NP size, we introduced PLGA, PEG-PLGA, or blended (PLGA+PEG-PLGA, m_PLGA_:m_PEG-PLGA_ = 5:5) acetonitrile solution (C_polymer_ = 5 mg/mL) with ultrapure water in the iLiNP device. The total flow rate (TFR) ranged from 50 to 500 μL/min, whereas the flow rate ratio (FRR) of aqueous phase to organic phase ranged from 3 to 9. Figs 2 and S1 show the NP sizes and size distributions. In the case of lipid nanoparticles (LNPs), small-sized LNPs formed under high TFR and FRR conditions [24–27]. The PLGA NP size also decreased with an increase in the TFR, maintaining a single peak. NP sizes were 44–101 nm, 29–76 nm, and 40–114 nm for PLGA, PEG-PLGA, and blend NPs, respectively. With restriction to changing fluid conditions, only a narrow NP size range (23–29 nm under 50 mg/mL concentration or 20–26 nm at 20 mg/mL concentration) could be achieved by Karnik’s method [23]. However, the FRR did not affect the NP size, which differs from a previous study [23]. PEG-PLGA induced a decrease in NP size, similar to PEG-DMG, in the LNP system. Blend NPs showed the widest NP size range among the three polymers. We also evaluated the effect of the PLGA concentration, molecular weight, and PLGA composition on the NP size, polydispersity index (PDI) and NP stability (S2–S5 Figs and S1 Table).

**Fig 3:**
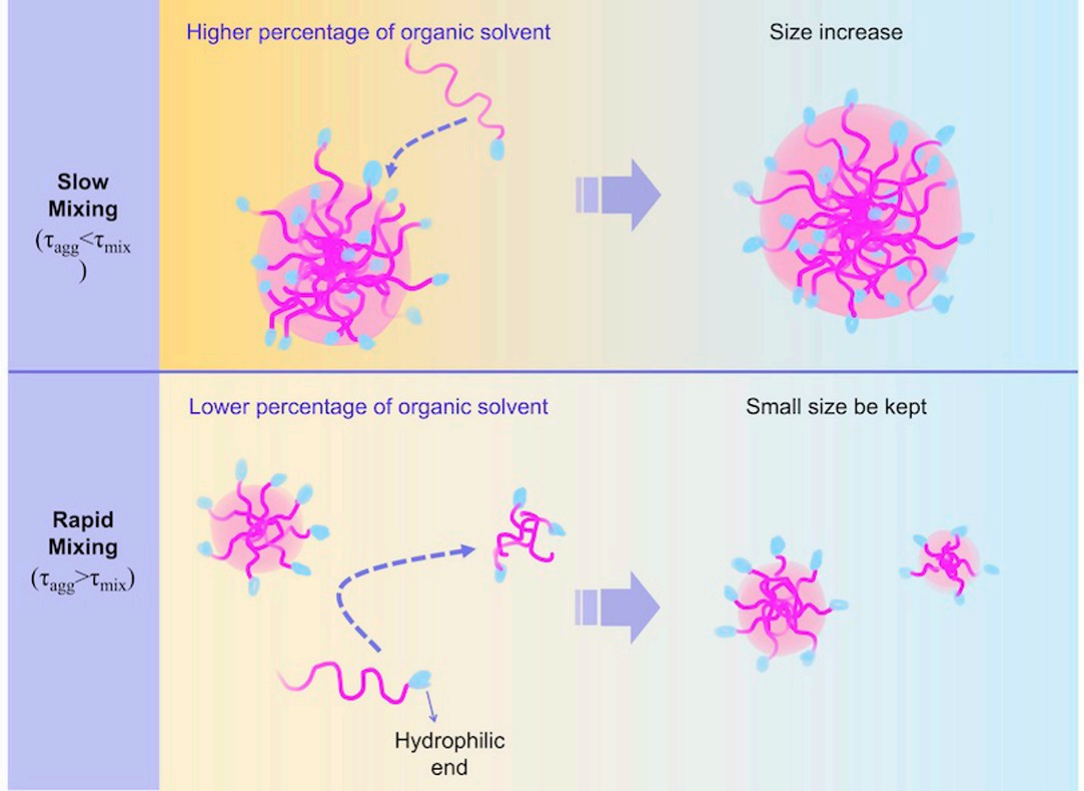
Mechanism of NP self-assembly during the solvent exchange. The relationship of the time scale of mixing and aggregation is the critical factor affecting the final particle size.

This NP formation behavior is probably due to the baffle structure of our device, which enables a far more effective solvent dilution performance than the sample flow-focusing device employed by Karnik. Based on the mechanism of self-assembly into microfluidic devices [23], the relationship between the solvent dilution time scale (*τ*_mix_) and the polymer aggregation time scale (*τ*_agg_) closely influences the size of the final NPs (Fig 3). In this study, the excellent solvent dilution performance allowed *τ*_mix_ to be much smaller than *τ*_agg_ in various FRR ranges. Therefore, polymer aggregation occurs when the solvent exchange is almost complete, and NP self-assembly occurs under conditions closer to the final solvent. Hence, the polymer does not readily assemble into NPs, resulting in smaller NPs. In contrast, the FRR change in this study cannot significantly affect the relationship between *τ*_mix_ and *τ*_agg_ and thus cannot significantly affect the NP size change. These results confirmed that the iLiNP device could control the NP size depending on the TFR without changing the precursors.

### Comparison of the iLiNP device with the conventional method/chaotic mixer device

**Fig 4:**
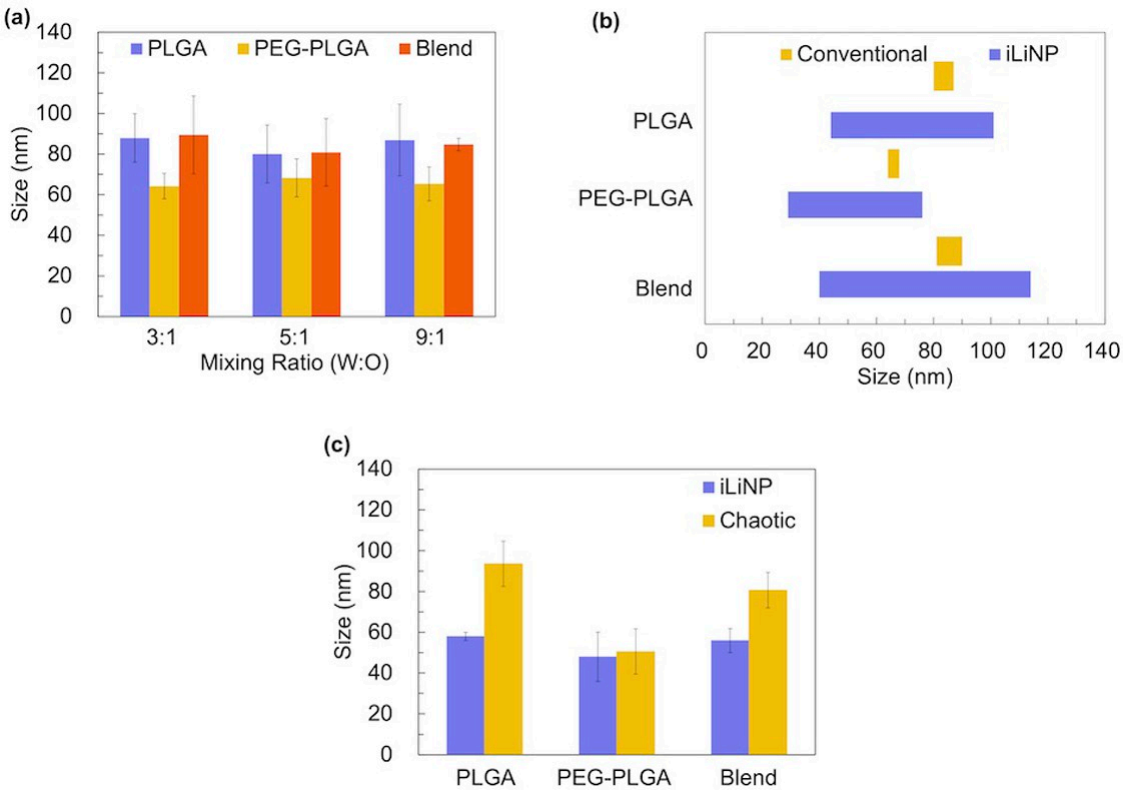
Comparison of NP sizes prepared using the iLiNP device, the chaotic mixer device, and the conventional method. (a) NP sizes prepared using the conventional method under different organic and aqueous solution ratios. The size range was: PLGA NPs: 80–87 nm; PEG-PLGA NPs: 64–68 nm; blend NPs: 81–90 nm; (b) Comparison of the particle size range of NPs prepared by the conventional method and the iLiNP device. (c) NP sizes prepared using the iLiNP device and the chaotic mixer device, NPs were prepared with FRR of 5 and TFR of 300 μL/min. The error bars represent the standard deviations calculated from the repeated NP preparation experiment more than three times.

We compared the PLGA NP formation behavior using the iLiNP, the conventional method, and the chaotic mixer device with only varying flow conditions. Based on the adjustable particle size range of NPs prepared by the conventional method shown in Fig 4A and the size range of NPs obtained from iLiNP preparation shown in Fig 2, we present the particle size range of the two methods as Fig 4B. From Fig 4B, it can be seen that the sizes of PLGA, PEG-PLGA, and blend NPs prepared by the conventional method ranging 80–87 nm, 64–68 nm, and 81–90 nm, respectively, whereas the NP sizes prepared using the iLiNP device ranged from 44 to 101 nm, 29 to 76 nm, and 40 to 114 nm, respectively. In addition, the iLiNP device showed good NP size reproducibility compared with the conventional method. The iLiNP device enables the preparation of a larger range of particle sizes than conventional preparation methods. The difference in NP formation behavior may be attributed to the solvent dilution performance of the iLiNP device. In contrast, in the conventional method, it is difficult to precisely control the solvent dilution speed (stirring speed corresponding to the TFR in the microfluidic method) manually on a macroscopic scale. This observation shows that PLGA NPs prepared using the iLiNP device demonstrate better size controllability and a broader range of sizes than those prepared by the conventional methods, which could supply various demands when using the same polymer to prepare NPs with a broad size distribution.

The chaotic mixer device [28] can effectively mix the solutions in the microchannel. The PLGA, PEG-PLGA, or blend acetonitrile solution at 5 mg/mL concentration was introduced into the chaotic mixer device with a TFR of 300 μL/min and FRR of 5. Under the same flow condition, the NPs prepared using the chaotic device were larger than those prepared using the iLiNP device, except PEG-PLGA NPs (Fig 4C). Based on this mechanism (Fig 3), *τ*_agg_ was observed to be larger than *τ*_mix_ at rapid dilution.

Karnik et al. compared the zeta potential results of nanoparticles prepared using the bulk method with those of the microfluidic device method by adding PLGA to pure PEG-PLGA with altered precursors [23]. The results indicated that the zeta potential of nanoparticles prepared using the bulk method increased substantially with the addition of PLGA, whereas the microfluidic method did not. The PEG chain segments have negatively charged carboxyl terminals, and the increase in zeta potential indicates that fewer PEG chain segments are exposed on the particle surface. Furthermore, combined with the comparison of the TFR effect within the microfluidic device, during rapid dilution, the polymer molecules are located in the solution environment in which the amount of organic solvent is smaller than that of water. At this point, the hydrophilic chain segment is almost not present inside the particle, and the surface of the hydrophilic PEG surface barrier is sufficient, making the absorption or insertion of polymers into the NPs difficult. Moreover, the excess polymers must undergo more nucleation. The inverse of this theory shows that more efficient and rapid dilution can result in smaller particle sizes. In addition, the similar size of PEG-PLGA NPs prepared by the chaotic mixer device and the iLiNP is most likely due to the fact that the proportion of PEG segment components in PEG-PLGA is more than that of PLGA or blend. More PEG segment means easier to form PEG protective shells quickly (*τ*_agg_ of PEG-PLGA is different from that of PLGA or blend). It is known from the mechanism of polymer self-assembly that when enough hydrophilic chain segments form a protective shell on the outside, it will not be possible to make more polymers insert into the NPs, thus not leading to larger particle size. Under the flow rate conditions compared in this section, likely, the difference in mixing rate between the two microfluidic devices at this time will not result in a significant change in the relationship between the *τ*_agg_ and *τ*_mix_ sizes of PEG-PLGA, and therefore the particle size will not change significantly.

In conclusion, these results demonstrate that the iLiNP device method can achieve a larger range of tunable NP size preparation than the bulk method and achieve rapid dilution of the organic solvent more efficiently than a typical microfluidic device chaotic mixer [24]. This finding demonstrates the superiority of this method and its great potential for further drug screening, custom drug delivery particle preparation, and other applications.

### Drug loading into NPs

**Fig 5:**
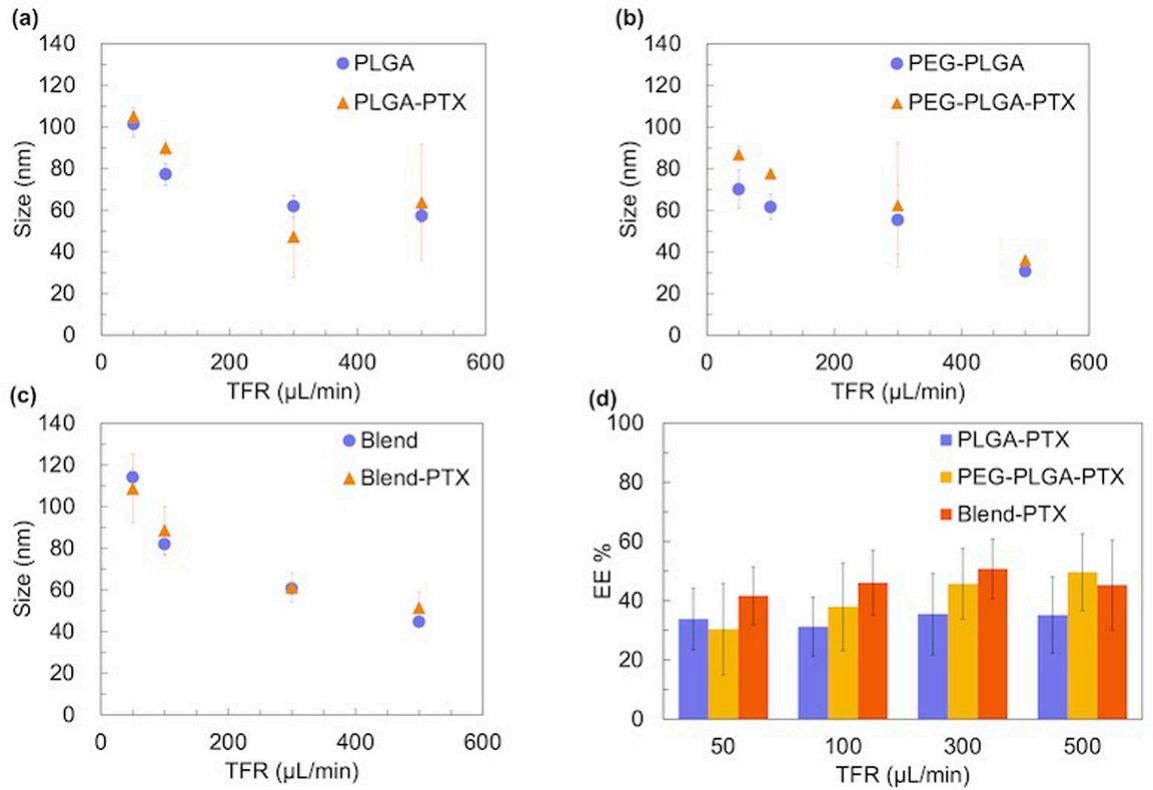
Size of blank NPs, PTX-loaded NPs, and EE of PTX. (a) PLGA NPs and PTX-loaded PLGA (PLGA-PTX) NPs; (b) PEG-PLGA NPs and PTX-loaded PEG-PLGA (PEG-PLGA-PTX) NPs; (c) blend NPs and PTX-loaded blend (blend-PTX) NPs. The purple dots represent blank NPs, and the orange dots are PTX-loaded NPs; (d) EE of PTX prepared at TFR of 50, 100, 300, and 500 μL/min and the FRR of 3. The error bars represent the standard deviations calculated from the repeated NP preparation experiment at least three times.

We loaded hydrophobic drugs into NPs using the iLiNP device and used PTX as a model drug. Fig 5A–5C shows a comparison between the average NP sizes of PTX-loaded NPs and unloaded-NPs prepared under different TFR conditions. Under the same flow condition of FRR = 3, PTX did not affect the NP size, regardless of the polymer type. The reproducibility of PTX-loaded NPs is slightly inferior to that of blank NPs (PTX-unloaded), which may be attributed to the hydrophobicity of PTX. The high hydrophobicity leads to more aggregation between particles during particle formation. This result proved that our method could be used to prepare blank and PTX-loaded NPs with a broad size range and good reproducibility. Moreover, the dependence of the PTX EE on the flow conditions and polymer composition was studied (Fig 5D). The EE was ranged from 30% to 70%, depending on the polymer type. PTX loaded PLGA (PLGA-PTX NPs) prepared using microfluidic devices always show low EE and loading capacity, which is attributed to the large amounts of drugs lost during solvent displacement, while the NPs formed by the polymer chain come together. This due to the principle of the continuous microfluidic method, which is the same as the solvent replacement method: while the preparation of drug-loaded PLGA NPs formed based on this principle, the drug is leaked out into the organic phase during solvent displacement [29–32]. Under the same polymer conditions, EE did not vary regularly with the TFR. Furthermore, the PTX-loaded blend (blend-PTX) NPs exhibited the highest EE among the three polymers. Compared to PTX-loaded PEG-PLGA (PEG-PLGA-PTX) NPs, the incorporation of PLGA increased EE, which is in agreement with a previous study [23]. This result is due to the hydrophobic PLGA increasing the hydrophobic core, thus improving the package capacity for the hydrophobic drug PTX. Meanwhile, PLGA-PTX NPs could not achieve a higher EE than blend-PTX NPs, which can be attributed to the lower PEG acting as hydrophobic layer protection for PLGA NPs. The loaded PTX, which is attached to the surface of PLGA NPs, is easily lost during the preparation process without the PEG brush barrier. Blend-PTX was selected for further *in vitro* experiments considering that blend-PTX NPs showed a larger NP size range than PLGA-PTX or PEG-PLGA-PTX NPs and enabled high EE.

### *In vitro* antitumor activity

**Fig 6:**
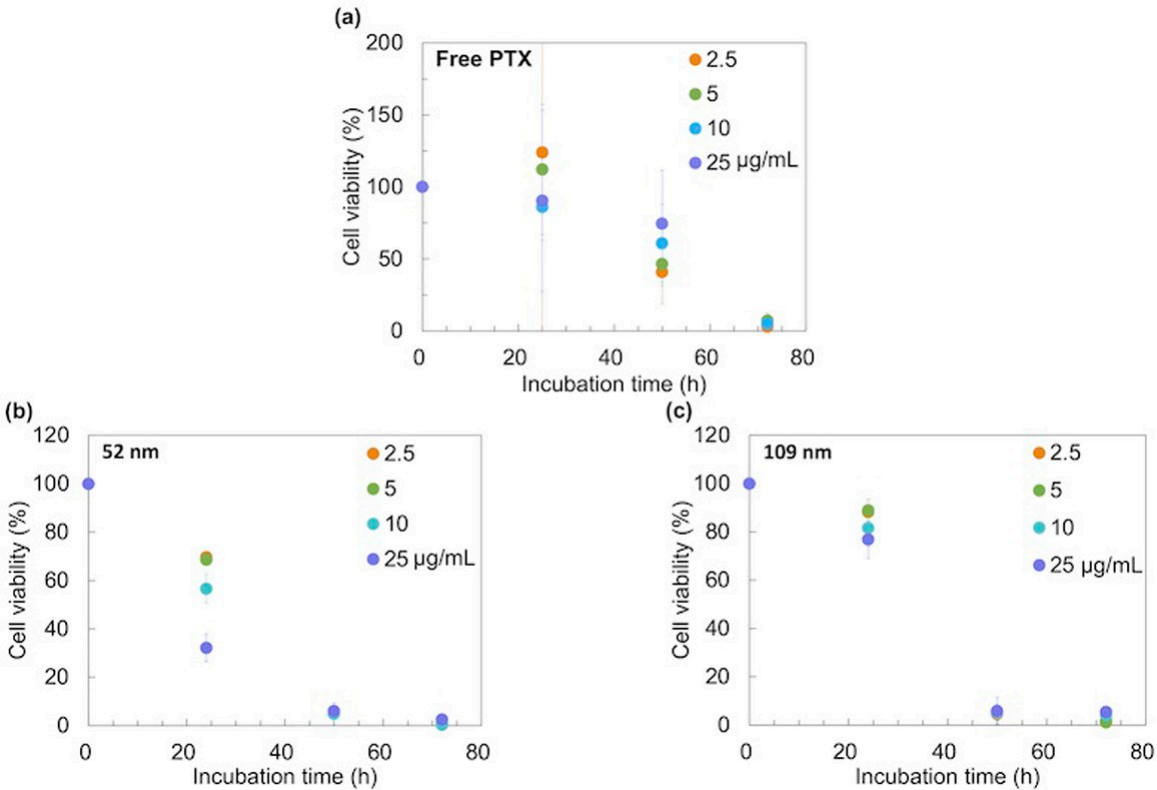
Cell viabilities dosed of (a) free-PTX, (b) 52 nm-sized blend-PTX NPs, and (c) 109 nm-sized blend-PTX NPs. Concentrations of PTX varied from 2.5 to 25 μg/mL. Preparation conditions for blend-PTX NPs: C_polymer_ = 5 mg/mL, TFR of 50 or 500 μL/min and the FRR of 3. The error bars represent the standard deviations calculated from the repeated NP preparation experiment at least three times.

To demonstrate the effect of NP size on cytotoxicity, we prepared 52 nm and 109 nm-sized blend-PTX NPs (The blend-PTX NPs properties are consistent with those obtained for the preparation at 50 and 500 μL/min in Fig 5C and 5D). Unlike other studies, the iLiNP device could control the NP size by TFR without changing the PLGA concentration, molecular weight, and composition. For the action mechanism of PTX, PTX binds microtubules and causes kinetic suppression of microtubule dynamics; thus, the cell cycle is consequently arrested at G2/M phases [33–35] We hypothesized that the NP size affects cellular uptake and PTX release kinetics and that this synergistic effect would affect cytotoxicity. Free-PTX, 52 nm-sized PTX-loaded NPs, and 109 nm-sized PTX-loaded NPs were added to HeLa cells at different dosages. Fig 6A shows the cell viability of free PTX unloaded into NPs. After 24 h, free PTX did not exhibit antitumor activity, regardless of the PTX concentration. Cell viability decreased to 50–80% and lower than 10% after 48 and 72 h of incubation, respectively. Fig 6B and 6C shows the antitumor activity of 52 and 109 nm-sized NPs. Cytotoxicity of the blank NPs (PTX-unloaded) were shown in S6 Fig. The blank blend NPs at 114 nm showed no toxicity from 0.1 μg/mL to 100 μg/mL, while the 45 nm blank blend NPs showed some toxicity to HeLa cells at higher concentrations. It has been previously reported that nano-sized PLGA particles can be toxic to cells to some extent [36, 37]. After incubation with 52 nm blend-PTX for 24 h, cell viability remarkably reduced, whereas 109 nm blend-PTX could not show significant cytotoxicity. After 50 h of incubation, cell growth was almost completely inhibited by PTX-loaded NPs, regardless of NP size, and no significant difference was found in cytotoxicity among the different concentrations. Furthermore, after 72 h of incubation, all PTX-loaded NPs completely inhibited cell growth. Based on these results, it was observed that the blend-PTX NPs with a 52 nm size could rapidly show high levels of antitumor activity compared to the 109 nm-sized NPs.

Previous studies have shown that the cellular uptake of different kinds of NPs, such as gold NPs, silver NPs, and mesoporous silica NPs, by HeLa cells depends on their size, with the maximum uptake occurring at 50 nm [38–41]. However, as mentioned earlier, studies on Caco-2 cancer cells showed that small particle size (50 nm) had less cellular uptake than larger particle size [14, 15]. In contrast, in KB cancer cells, small NP size (70 nm) had more cellular uptake than larger particles. This contrast is may be due to the different cell types, indicating that even for cancer cells, different cancer cell lines show different cellular uptake based on particle sizes. The effect of polymer NP size on the same precursor preparation of HeLa cells has not been published, and our results precisely complement the research gap in this regard. In addition, our findings demonstrate a stronger case for providing a more finely size turnable PLGA NPs and satisfy the need to study the particle size effect and the mechanism behind it for targeted drug therapy.

## Conclusion

In this study, size-tunable PLGA NPs were prepared using the iLiNP device. High flow rate produced small-sized NPs, whereas FRR did not markedly impact the size. In addition, the iLiNP device can prepare PLGA-based NPs with a broader size range (PLGA NPs: 44–101 nm; PEG-PLGA NPs: 29–76 nm; blend NPs: 40–114 nm) than those prepared using the conventional bulk method (PLGA NPs: 80–87 nm; PEG-PLGA NPs: 64–68 nm; blend NPs: 81–90 nm) and chaotic mixer device. In addition, PTX was loaded into PLGA/PEG-PLGA/blend NPs. We found that 52 nm-sized PTX-loaded NPs inhibited cell growth and showed higher antitumor activity than 109 nm-sized NPs. The NPs were prepared using the same polymer solution under different TFR conditions. An NP size-tunable technique without any optimization of molecular weight, concentration, and composition of the polymer is crucial for developing DDS nanomedicines. We believe that iLiNP-based polymer-based NP production will provide abundant possibilities for future clinical applications of size-controlled nanomedicines.

## Supporting information

S1 Fig Size distribution of FRR = 5 with different TFR (μL/min). (a) PLGA, (b) PEG-PLGA, and (c) Blend.
(TIF)

S2 Fig Effect of PLGA concentration. NPs were prepared at (a) TFR = 300 μL/min and (b) TFR = 500 μL/min. Concentration of PLGA acetonitrile solution varying from 2.5 to 10 mg/mL. The error bars represent the standard deviations calculated from repeated NP preparation experiments at least three times.
(TIF)

S3 Fig Effect of PLGA Mw. (a) TFR = 300 μL/min and (b) TFR = 500 μL/min.
(TIF)

S4 Fig Effect of polymer composition. (a) Size and polydispersity index (PDI) comparison and (b) Size distribution with different PLGA:PEG-PLGA ratios. Data are presented as mean ± SEM; N > 3. We examined the effect of polymer composition on NPs using acetonitrile to dissolve different mass ratios of PLGA(M) with PEG-PLGA at 5 mg/mL concentration, TFR = 300 μL/min, and FRR = 5. The ratio of PLGA to PEG-PLGA ranged from 0:10 to 10:0; in this case, 0:10 indicates neat PEG-PLGA, and 10:0 indicates neat PLGA. The NP size decreased from 80 ± 6 nm to 43 ± 2 nm with the increase in PEG-PLGA concentration in blends (S4 Fig). This result may be attributed to the hydrophilic PEG blocks. Nucleation is achieved after the first stage of polymer self-assembly into NPs, and the unimers add to the nucleus. Unlike neat PLGA, after the polymer brush layer is formed on the particle surface, the hydrophilic PEG block of PEG-PLGA acts as a shell of particles, which can increase the barrier to avoid aggregation; consequently, the size is smaller than that of the neat PLGA NPs. This result indicates that the polymer composition plays an important role in the preparation of small-sized NPs, which is consistent with other studies.
(TIF)

S5 Fig Evaluation of NP stability. (a) PLGA, (b) PEG-PLGA, and (c) blended NPs stored at 4°C (purple dot) or 25°C (orange dot). Data are presented as mean ± SEM; N = 3. In addition to particle size and size distribution, the stability of NPs is significant both in vitro and in vivo. Ensuring the stability of the polymeric NPs during the long-term storage transportation would facilitate its effect. To check the stability of the NPs, the prepared PLGA NPs, PEG-PLGA NPs, and the blend were stored for 20 d at 4°C and 25°C. The particle size was measured at predetermined time intervals. All NP types showed no significant differences during 20 d (S5 Fig) and maintained a small size. In addition, NPs combined with PLGA showed slightly weaker stability than those without PLGA because the PEG layer acts as a shell around particles and reduces their interactions with foreign molecules, which can enhance the stability of particles. This result proved that the PLGA-based NPs prepared by the baffle device maintain high stability before uptake.
(TIF)

S6 Fig *In vitro* blank blended NP cell viability in HeLa cells. The blend concentration varying from 0.1 μg/mL to 100 μg/mL. (a) the average size of NPs is 45 nm, prepared at TFR = 500 μL/min. (b) the average size of NPs is 114 nm, prepared at TFR = 50 μL/min. Data are presented as mean ± SEM; N > 3. The cytotoxicity of blank blended NPs was investigated. The 114 nm blank NPs showed no cytotoxicity in HeLa cells, indicating that the toxicity of blended NPs I s mainly caused by the captured PTX and not the blank NPs. In contrast, the 45 nm-sized blended NPs showed no cytotoxicity to cells at low concentrations; however, it would enable cytotoxicity when the concentration is high (100 μg/mL).
(TIF)

S1 Table Formulation code. (TIF)
