# Supplementary figures and images for: Preparation of size-tunable sub-200 nm PLGA-based nanoparticles with a wide size range using a microfluidic platform
Source: PLoS One. 2022 Aug 4;17(8):e0271050. doi: 10.1371/journal.pone.0271050 (PMC9352036; doi:10.1371/journal.pone.0271050)

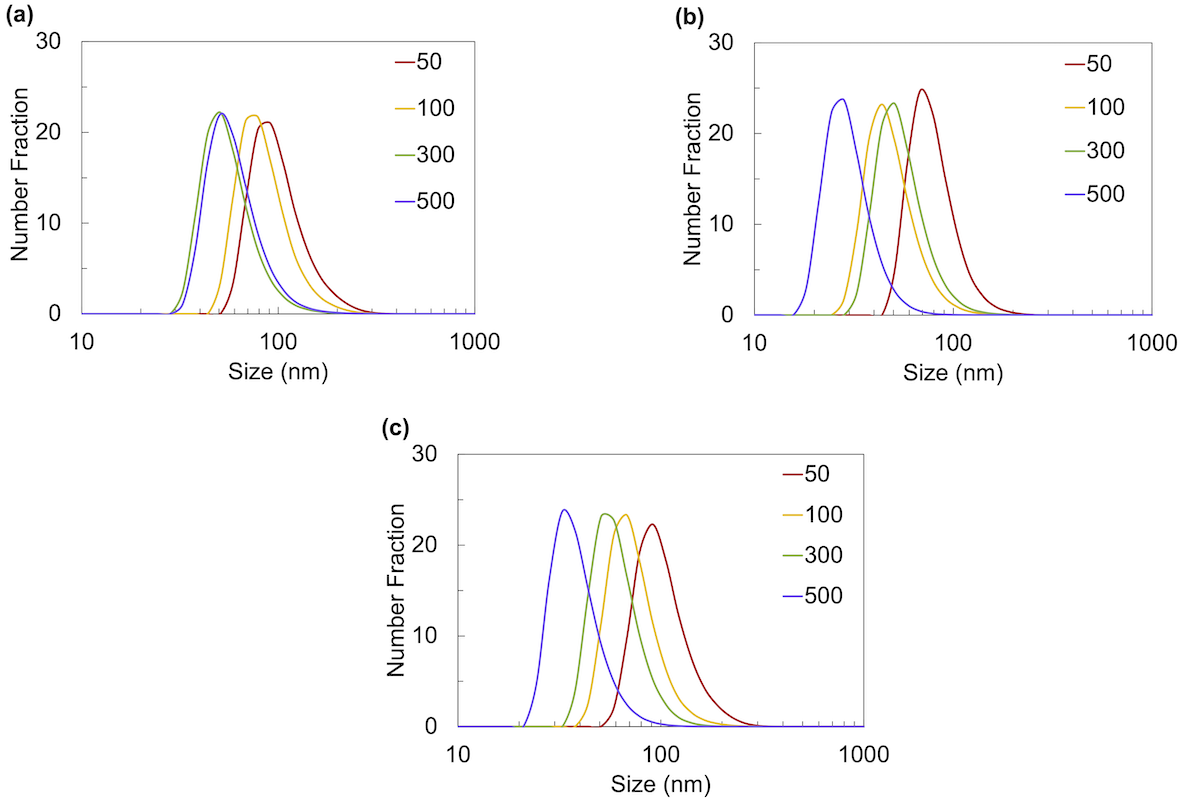

Supplement: S1 Fig — (a) PLGA, (b) PEG-PLGA, and (c) Blend. (TIF) [file pone.0271050.s001.tif]

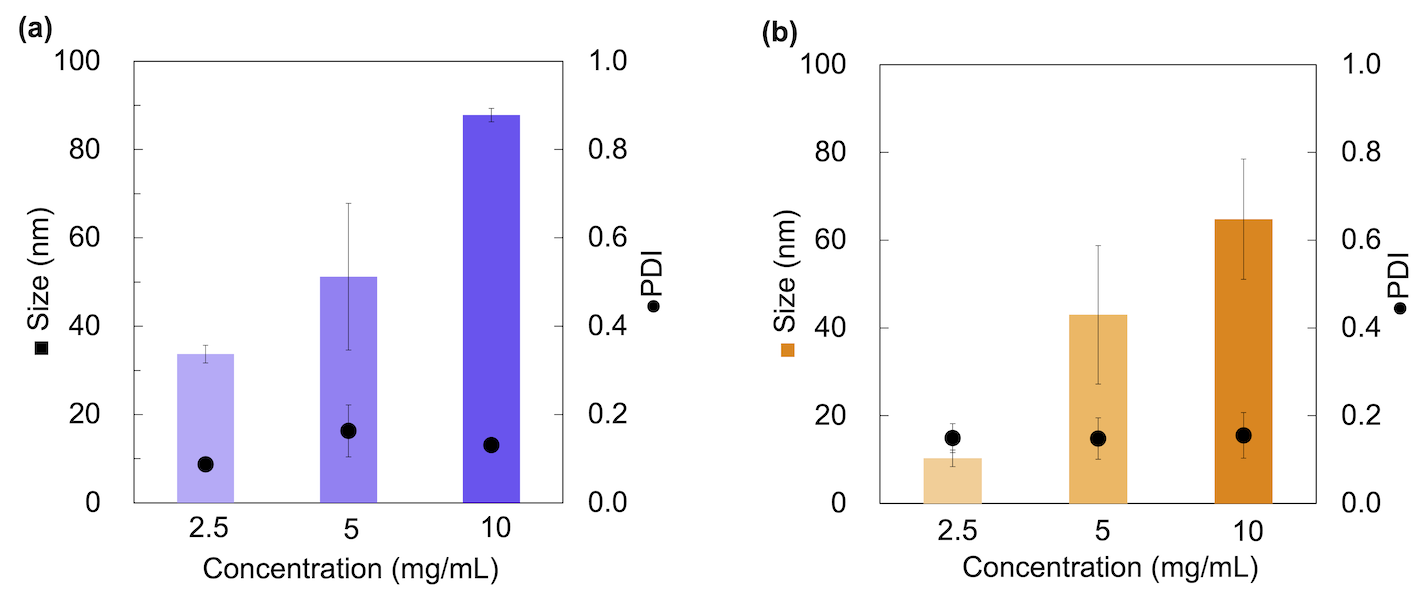

Supplement: S2 Fig — NPs were prepared at (a) TFR = 300 μL/min and (b) TFR = 500 μL/min. Concentration of PLGA acetonitrile solution varying from 2.5 to 10 mg/mL. The error bars represent the standard deviations calculated from repeated NP preparation experiments at least three times. (TIF) [file pone.0271050.s002.tif]

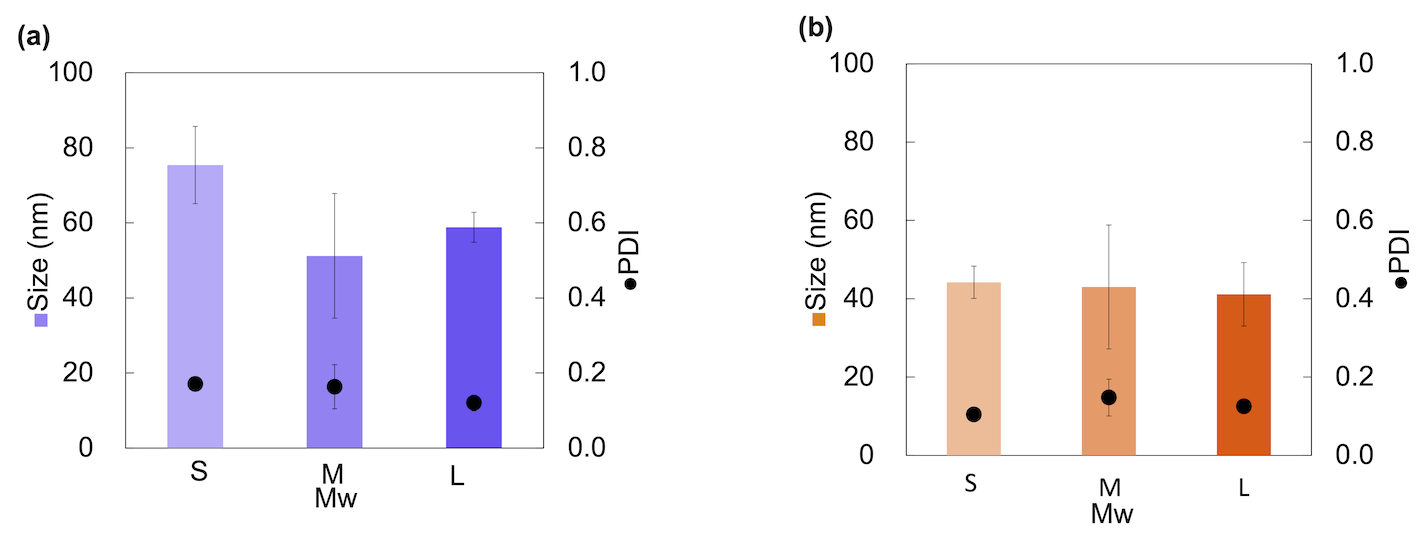

Supplement: S3 Fig — (a) TFR = 300 μL/min and (b) TFR = 500 μL/min. (TIF) [file pone.0271050.s003.tif]

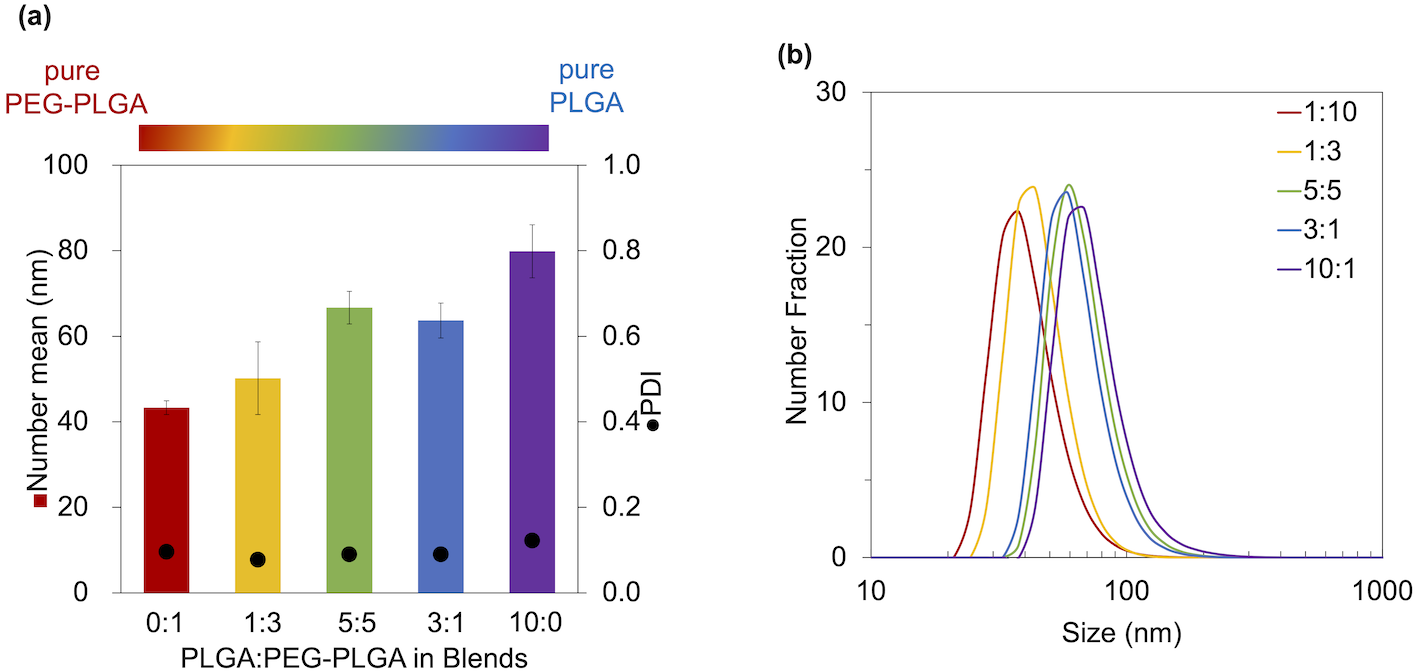

Supplement: S4 Fig — (a) Size and polydispersity index (PDI) comparison and (b) Size distribution with different PLGA:PEG-PLGA ratios. Data are presented as mean ± SEM; N > 3. We examined the effect of polymer composition on NPs using acetonitrile to dissolve different mass ratios of PLGA(M) with PEG-PLGA at 5 mg/mL concentration, TFR = 300 μL/min, and FRR = 5. The ratio of PLGA to PEG-PLGA ranged from 0:10 to 10:0; in this case, 0:10 indicates neat PEG-PLGA, and 10:0 indicates neat PLGA. The NP size decreased from 80 ± 6 nm to 43 ± 2 nm with the increase in PEG-PLGA concentration in blends (S4 Fig). This result may be attributed to the hydrophilic PEG blocks. Nucleation is achieved after the first stage of polymer self-assembly into NPs, and the unimers add to the nucleus. Unlike neat PLGA, after the polymer brush layer is formed on the particle surface, the hydrophilic PEG block of PEG-PLGA acts as a shell of particles, which can increase the barrier to avoid aggregation; consequently, the size is smaller than that of the neat PLGA NPs. This result indicates that the polymer composition plays an important role in the preparation of small-sized NPs, which is consistent with other studies. (TIF) [file pone.0271050.s004.tif]

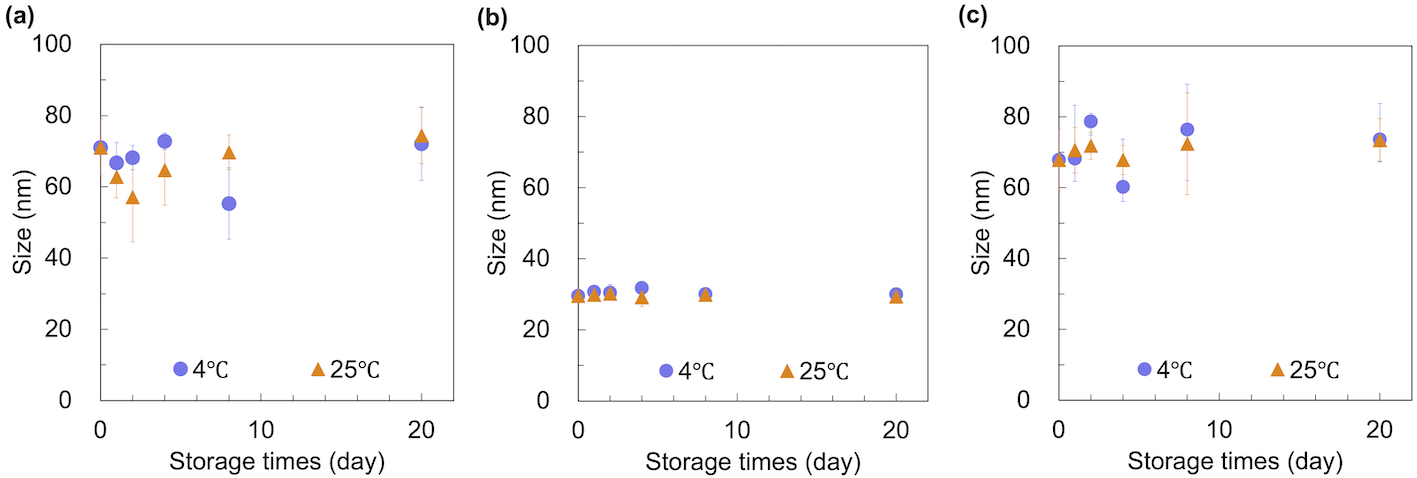

Supplement: S5 Fig — (a) PLGA, (b) PEG-PLGA, and (c) blended NPs stored at 4°C (purple dot) or 25°C (orange dot). Data are presented as mean ± SEM; N = 3. In addition to particle size and size distribution, the stability of NPs is significant both in vitro and in vivo. Ensuring the stability of the polymeric NPs during the long-term storage transportation would facilitate its effect. To check the stability of the NPs, the prepared PLGA NPs, PEG-PLGA NPs, and the blend were stored for 20 d at 4°C and 25°C. The particle size was measured at predetermined time intervals. All NP types showed no significant differences during 20 d (S5 Fig) and maintained a small size. In addition, NPs combined with PLGA showed slightly weaker stability than those without PLGA because the PEG layer acts as a shell around particles and reduces their interactions with foreign molecules, which can enhance the stability of particles. This result proved that the PLGA-based NPs prepared by the baffle device maintain high stability before uptake. (TIF) [file pone.0271050.s005.tif]

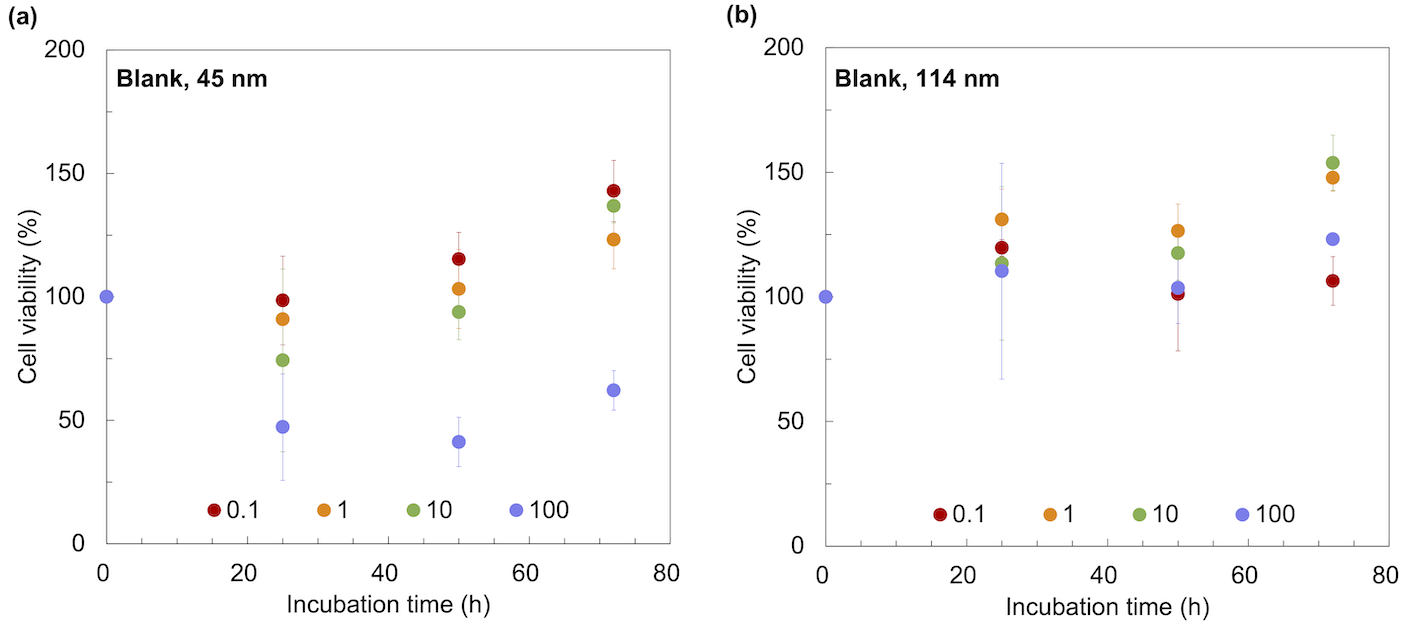

Supplement: S6 Fig — The blend concentration varying from 0.1 μg/mL to 100 μg/mL. (a) the average size of NPs is 45 nm, prepared at TFR = 500 μL/min. (b) the average size of NPs is 114 nm, prepared at TFR = 50 μL/min. Data are presented as mean ± SEM; N > 3. The cytotoxicity of blank blended NPs was investigated. The 114 nm blank NPs showed no cytotoxicity in HeLa cells, indicating that the toxicity of blended NPs I s mainly caused by the captured PTX and not the blank NPs. In contrast, the 45 nm-sized blended NPs showed no cytotoxicity to cells at low concentrations; however, it would enable cytotoxicity when the concentration is high (100 μg/mL). (TIF) [file pone.0271050.s006.tif]

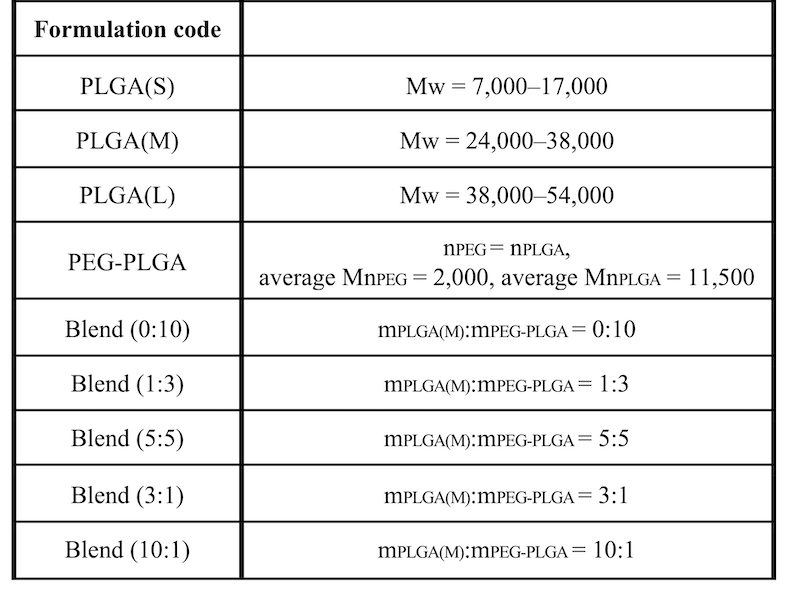

Supplement: S1 Table — (TIF) [file pone.0271050.s007.tif]
